# Utility of multimodal deep learning model to diagnose lymph node metastasis in esophageal cancer using computed tomography and positron emission tomography images

**DOI:** 10.1007/s00595-025-03152-5

**Published:** 2025-10-18

**Authors:** Yasuharu Shinozaki, Hirotaka Ishida, Tomofumi Kaneno, Eichi Takaya, Tomoya Kobayashi, Yusuke Taniyama, Chiaki Sato, Hiroshi Okamoto, Yohei Ozawa, Takuya Ueda, Takashi Kamei

**Affiliations:** 1 Department of Surgery, Tohoku University Graduate School of Medicine, 1-1 Seiryo-Machi, Aoba-Ku, Sendai, Miyagi 980-8574 Japan; 2 Department of Diagnostic Imaging, Tohoku University Graduate School of Medicine, Sendai, Japan; 3 AI Lab, Tohoku University Hospital, Sendai, Japan; 4 Department of Diagnostic Radiology, Tohoku University Graduate School of Medicine, Sendai, Japan

**Keywords:** Computed tomography, Esophageal cancer, Lymph node metastasis, Multimodal deep learning model, Positron emission tomography

## Abstract

**Purpose:**

This study aimed to assess the performance of a deep learning model using multimodal imaging for detecting lymph node metastasis in esophageal cancer in comparison to expert assessments.

**Methods:**

A retrospective analysis was performed for 521 lymph nodes from 167 patients with esophageal cancer who underwent esophagectomy. Deep learning models were developed based on multimodal imaging, including non-contrast-enhanced computed tomography, contrast-enhanced computed tomography, and positron emission tomography imaging. The diagnostic performance was evaluated and compared with expert assessments using a receiver operating characteristic curve analysis.

**Results:**

The area under the receiver operating characteristic curve values for the deep learning model were 0.81 with multimodal imaging, 0.73 with non-contrast-enhanced computed tomography, 0.72 with contrast-enhanced computed tomography, and 0.75 with positron emission tomography were calculated. The area under the curve of the deep learning model (0.81) demonstrated diagnostic performance comparable to that of experienced experts (area under the curve, 0.84; *P* = 0.62, DeLong’s test).

**Conclusion:**

The multimodal deep learning model using computed tomography and positron emission tomography demonstrated performance comparable to that of experts in diagnosing the presence of lymph node metastasis, a key prognostic factor in esophageal cancer, suggesting its potential clinical utility.

**Supplementary Information:**

The online version contains supplementary material available at 10.1007/s00595-025-03152-5.

## Introduction

Esophageal cancer is the sixth leading cause of cancer-related mortality worldwide, accounting for 5.3% of all cancer-related deaths [1, 2]. Lymph node metastasis serves as a critical prognostic factor in esophageal cancer [3–6]; therefore, accurate assessment of lymph node involvement is essential. Moreover, the diagnosis of lymph node metastasis significantly influences treatment planning, including decisions regarding the administration of neoadjuvant chemotherapy [7–9].

Computed tomography (CT) and positron emission tomography (PET)/CT are considered essential imaging modalities in the pretreatment evaluation of esophageal cancer. On CT imaging, lymph nodes exceeding 1 cm in diameter are generally considered positive for lymph node metastasis, with 56.0% sensitivity, 97.3% specificity, and 92.4% accuracy [10]. On PET/CT, lymph nodes demonstrating the uptake of ^18^F-fluorodeoxyglucose (FDG) were classified as positive for lymph node metastasis, with 60.7% sensitivity, 99.5% specificity, and 94.8% accuracy [10]. According to Lee et al., a combined interpretation using a standardized uptake value of > 2.6 along with iso- or low-attenuation on CT, yielded significantly improved diagnostic performance in detecting malignant lymph nodes in esophageal cancer [11]. However, inaccuracies in the diagnosis of lymph node metastasis result in missed opportunities for appropriate treatment. Therefore, further improvements in the diagnostic accuracy of lymph node assessments are necessary.

In recent years, deep learning (DL)-based artificial intelligence (AI) systems have evolved rapidly within the medical field, particularly for the analysis of diagnostic imaging. Convolutional neural networks, as a core architecture of DL models, have been utilized efficiently for image detection and classification by efficiently learning image features [12]. These models have been applied across various medical domains such as skin cancer classification [13] and diabetic retinopathy detection [14]. In gastroenterology, DL models have been employed for endoscopic imaging of gastrointestinal cancers [15, 16]. Takeuchi et al. developed a DL-based system for the identification of esophageal cancer using CT imaging [17]. Although DL models have been explored for the prediction of lymph node metastasis in lung and breast cancers [18, 19], no study has addressed their application in the diagnosis of lymph node metastasis in esophageal cancer. Furthermore, multimodal models, AI approaches that integrate multiple imaging modalities, have been introduced for medical diagnostics. Previous studies have demonstrated that such models provide superior diagnostic performance in comparison to single-modality models, particularly in the assessment of glioma and breast cancers [20, 21].

Therefore, the present study aimed to (1) develop a DL model for the diagnosis of lymph node metastasis using non-contrast-enhanced CT (nCECT), contrast-enhanced CT (CECT), and PET images and (2) evaluate the utility of a DL-based multimodal image analysis method.

## Methods

### Patient data

**Fig. 1:**
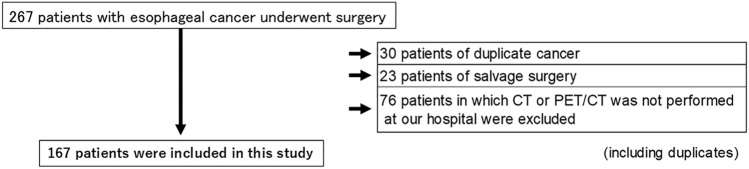
Study flowchart. This study included 267 patients with esophageal cancer who underwent esophagectomy. Of these, 30 patients with synchronous cancer, 23 who underwent salvage surgery, and 76 for whom CT or PET/CT imaging had not been performed at the study institution were excluded (including duplicate cases). Finally, 167 patients were included in the final analysis. *CT*, computed tomography; *PET*, positron emission tomography

This study included 267 patients with esophageal cancer who underwent esophagectomy at Tohoku University Hospital between January 2017 and December 2020. Among these, 30 patients with synchronous or metachronous cancers, 23 who underwent salvage surgery, and 76 who did not undergo CT or PET/CT imaging at the institution were excluded (including duplicate cases). Consequently, 167 patients were enrolled in this study (Fig. 1). For patients who received neoadjuvant treatment before esophagectomy, imaging data obtained before the initiation of neoadjuvant treatment were used. Lymph nodes dissected during surgery were classified and numbered in accordance with the Japanese Classification of Esophageal Cancer, 12th Edition [22, 23]. If two or more lymph nodes were detected in the same anatomical region, the largest node was designated as the representative node [22, 23]. Lymph nodes measuring less than 5 mm in diameter, which could not be reliably detected by PET/CT, were excluded [24]. Furthermore, lymph nodes that could not be clearly distinguished from primary tumors were excluded. Based on these criteria, 521 lymph nodes were included in the final analysis. The presence of lymph node metastasis was confirmed by a histopathological examination of surgical specimens.

### Preoperative adjuvant therapy and surgical procedures

In accordance with the findings of the JCOG9907 trial and Esophageal Cancer Practice Guidelines 2017, preoperative chemotherapy with cisplatin and 5-fluorouracil was administered to patients diagnosed with clinical stage II or III esophageal cancer [8, 25]. Additionally, preoperative chemoradiotherapy was performed in selected patients with locally advanced disease.

On the day of surgery, methylprednisolone (250 mg) was administered intravenously in the morning. Thoracoscopic or robot-assisted minimally invasive esophagectomy combined with radical lymphadenectomy was performed using the McKeown procedure, with patients in the left semi-prone position [26–28]. The extent of lymph node dissection was determined based on the tumor location, preoperative clinical stage, and individual patient factors. During the abdominal procedure, upper abdominal lymphadenectomy was performed, and the gastric conduit was prepared using hand-assisted laparoscopic surgery, open laparotomy, laparoscopy, or robot-assisted techniques, as previously described [26–28]. The gastric conduit was subsequently elevated through either the posterior mediastinal or retrosternal route, and cervical esophagogastric anastomosis was performed using Gambee sutures or a mechanical technique with linear staplers.

### Image data

nCECT and CECT images were acquired using the Somatom Definition AS scanner (Siemens Healthcare, Erlangen, Germany) and Aquilion ONE system (Canon, Tokyo, Japan). For the contrast-enhanced study, imaging was performed 90 s after intravenous administration of the contrast medium at a rate of 4 mL/s. CT images were reconstructed with a section thickness of 1 mm and matrix size of 512 × 512. PET/CT images were acquired using Discovery MI (GE Healthcare, Chicago, IL, USA) and Biograph-40 (Siemens Healthcare) scanners. Prior to imaging, patients underwent a minimum fasting period of 4 h, followed by intravenous administration of ^18^F-FDG at a dose of 3.75 MBq/kg, and were rested for 1 h. PET imaging of the corresponding anatomical region was performed in three-dimensional mode at 2 min per bed position, with bed position increments of 20 cm for the Discovery MI and 16.2 cm for the Biograph-40 systems. The PET images were attenuation-corrected and reconstructed using an ordered-subset expectation maximization iterative algorithm, in accordance with the manufacturer’s specifications, resulting in final pixel sizes of 4.2 mm and 4.1 mm for the Discovery MI and Biograph-40, respectively.

To standardize the image dimensions, lymph node regions were cropped to 32 × 32 mm, resized to 512 × 512 pixels, and reconstructed in an 8-bit grayscale format. A total of 521 lymph node images were randomly divided into two subsets: a training dataset including 80% of the images (n = 416) that was used to train the DL model, and a test dataset including the remaining 20% (n = 105) that was reserved for model evaluation. For the 105 lymph nodes included in the test dataset, the predictive probability of metastasis was calculated using the DL model trained on the corresponding training data.

### Image interpretation by experts

Metastatic lymph nodes are generally characterized by the following imaging features: (1) a short axis diameter greater than 10 mm and a short-to-long axis ratio > 0.67 nCECT, (2) a short axis diameter of 10 mm or more, a short-to-long axis ratio > 0.67, and the presence of contrast enhancement on CECT; and (3) a standardized uptake value of at least 2.6 on PET/CT [11, 29–31]. To compare the diagnostic performance for lymph node metastasis, nCECT, CECT, and PET were independently assessed by three board-certified esophageal surgeons accredited by the Japan Esophageal Society, each with > 15 years of experience in the diagnosis and treatment of esophageal cancer, including imaging interpretation. A quasi-continuous confidence rating scale, ranging from 0 to 100 (0 = not at all confident of metastasis; 100 = completely confident of metastasis), was used to evaluate each imaging modality. In accordance with clinical practice, evaluation was conducted using multimodal images that included nCECT, CECT, and PET scans. The experts-reviewed images were identical to those obtained using the DL model. Each expert provided confidence scores based on the integrated assessment of all three modalities, and the mean value of the three experts’ scores was used for all the analyses. The likelihood of lymph node metastasis was assessed using a receiver operating characteristic (ROC) curve analysis based on the mean value of the quasi-continuous confidence scores provided by the three experts.

### DL model

**Fig. 2:**
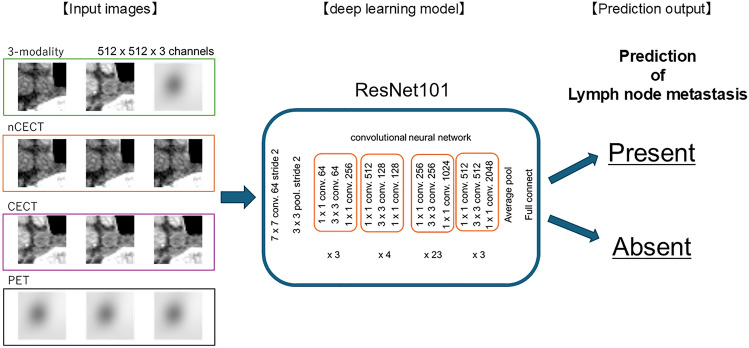
Schematic illustration of the deep learning model used in this study, In the dataset incorporating the three imaging modalities, images from each modality were assigned to one of the three input channels and processed using a residual neural network (ResNet101) for the diagnosis of lymph node metastasis in esophageal cancer. n*CECT* non-contrast-enhanced computed tomography, *PET* positron emission tomography, *CECT* contrast-enhanced computed tomography

A DL model was developed to predict the probability of lymph node metastasis using Python (ver. 3.8.2, Python Software Foundation, http://www.python.org) and PyTorch (ver. 1.5.1) as the DL framework. Four models were constructed: three single-modality models trained separately using nCECT, CECT, or PET and one multimodal model that integrated all three imaging modalities. For the multimodal DL model, images from the three modalities were assigned to separate input channels and simultaneously input into the ResNet101 architecture for lymph node classification [32]. The ResNet101 model was pre-trained on the ImageNet dataset. Model training was performed using the Adam optimizer with a learning rate of 0.000874 and a weight decay of 0.000001. A categorical cross-entropy loss function was applied, and the batch size was set to 32. The optimal number of training epochs was determined using three-fold cross-validation, and was set to 50, 26, 16, and 17 for the nCECT, CECT, PET, and multimodal models, respectively (Fig. 2).

The system was implemented on a computer equipped with an Intel Core i7-7800X CPU (six cores) and an NVIDIA QUADRO RTX 8000 Graphics Processing Unit with 48 GB memory. The operating system was Ubuntu (ver. 16.04.5; Long-Term Support: Xenial Xerus).

### Statistical analysis

Differences in clinicopathological factors and immunoreactivity were evaluated using the χ^2^ test. The performance of DL models was assessed based on the area under the curve (AUC), sensitivity, specificity, and accuracy. To calculate sensitivity, specificity, and accuracy, the cutoff value for the quasi-continuous confidence rating scale was set to 50 [33]. The statistical significance of the differences among the ROC curves of the single-modality DL models, multimodal DL model, and expert assessments was evaluated using DeLong’s test, with *P*-values of < 0.05 considered to indicate statistical significance. All statistical analyses were performed using Scikit-learn (ver. 1.0.2; https://scikit-learn.org).

### Ethics

This study was approved by the Ethics Committee of Tohoku University School of Medicine (2021–1–623) and was conducted in accordance with the ethical standards outlined in the 1964 Declaration of Helsinki and its later amendments. Comprehensive consent for participation and publication was obtained from all patients. Owing to the retrospective nature of this study, an opt-out method was employed by disclosing the study information on the institutional website.

## Results

### Patient and lymph node characteristics

The clinicopathological factors of the 167 patients included in this study are summarized in Online Resource 1. Among the 521 lymph nodes analyzed, 150 (28.8%) were confirmed to have metastatic lesions. The anatomical distribution of primary tumors with corresponding lymph node information was as follows (non-metastatic: metastatic): cervical esophagus, 2:1; upper thoracic esophagus, 12:8; middle thoracic esophagus, 179:56; lower thoracic esophagus, 112:44; and abdominal esophagus, 66:41. The distribution of pretreatment T-factors with lymph node status was as follows (non-metastatic: metastatic): T1a, 25:1; T1b, 122:19; T2, 64:25; T3, 151:99; and T4a, 9:6 (Table 1). The proportion of lymph node metastases was identical between the training dataset (*n* = 416) and the test dataset (*n* = 105), as shown in Table 1.

**Table 1:** Characteristics of lymph nodes analyzed in this study

	All data		Test data		*P*
	Lymph node metastasis (N)		Lymph node metastasis (N)		
	Absent	Present	Absent	Present	
Tumor location					
Cervical	2	1	1	0	
Upper thoracic	12	8	2	1	
Middle thoracic	179	56	29	12	
Lower thoracic	112	44	27	10	
Abdominal	66	41	16	7	0.846
Histology					
Squamous cell carcinoma	268	106	50	20	
Adenocarcinoma	68	44	17	10	
Others	35	0	8	0	0.775
Tumor depth					
T1a	25	1	4	0	
T1b	122	19	24	3	
T2	64	25	11	3	
T3	151	99	34	24	
T4a	9	6	2	0	0.782
Total	371 (71.2%)	150 (28.8%)	75 (71.4%)	30 (28.6%)	

### Diagnostic performance for lymph node metastasis by the DL model and experts

**Fig. 3:**
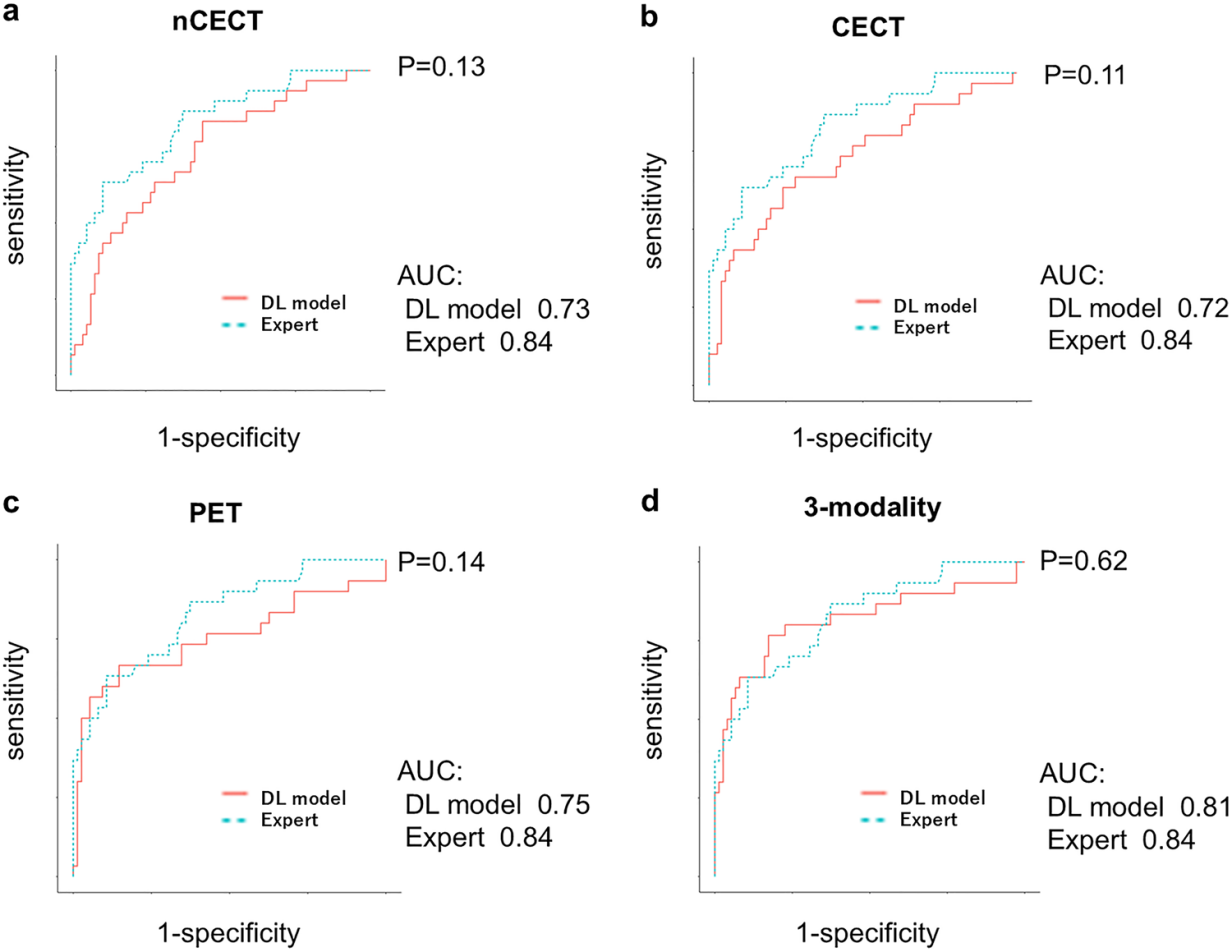
Comparison of the area under the curve for each modality and multimodal approach, assessed by the deep learning model and experts. The AUC values of nCECT, CECT, and PET for the diagnosis of lymph node metastasis using the DL model were 0.73, 0.72, and 0.75, respectively, whereas the AUC value of the multimodal DL model incorporating all three imaging modalities was 0.81. The AUC values obtained by the experts (multimodal) and the single-modality DL model are presented in Fig. 3a–c (nCECT, *P* = 0.13; CECT, *P* = 0.11; PET, *P* = 0.14). Furthermore, the multimodal DL model incorporating all three imaging modalities demonstrated comparable performance to the experts (AUC: multimodal DL model, 0.81; expert, 0.84; *P* = 0.62) (Fig. 3 d). In accordance with clinical practice, expert assessments were performed using multimodal images, including nCECT, CECT, and PET scans. *AUC* area under the curve, *DL* deep learning, *nCECT* non-contrast-enhanced computed tomography, *CECT* contrast-enhanced computed tomography, *PET* positron emission tomography

The AUCs for the diagnosis of lymph node metastasis using the DL model based on the nCECT, CECT, and PET images were 0.73, 0.72, and 0.75, respectively. By contrast, the multimodal DL model incorporating all three imaging modalities achieved an AUC of 0.81 (Fig. 3). Although the differences in the AUCs between the single-modality DL models and multimodal DL model were not significant (nCECT, *P* = 0.21; CECT, *P* = 0.19; PET, *P* = 0.28), only the multimodal DL model achieved an AUC of > 0.80. The AUC of the multimodal image assessment performed by the experts was 0.84 (Fig. 3). The sensitivity, specificity, and accuracy of the single-modality DL models, multimodal DL model, and expert evaluations are summarized in Table 2. When the three imaging modalities were integrated, the multimodal DL model exhibited comparable diagnostic performance to the experienced specialists. Notably, the multimodal DL model achieved 60% sensitivity, which exceeded the sensitivity observed among experts (~ 37%). The AUCs for expert assessment (performed using multimodal images in accordance with clinical practice) and each of the DL single-modality models are presented in Fig. 3a–c (nCECT, *P* = 0.13; CECT, *P* = 0.11; PET, *P* = 0.14). Furthermore, the multimodal DL model incorporating all three imaging modalities demonstrated comparable performance to the expert interpretation (AUC: multimodal DL model, 0.81; expert, 0.84; *P* = 0.62) (Fig. 3d).

**Table 2:** Comparison of diagnostic performance between the deep learning model and experts

	Deep learning model				Expert
	nCECT	CECT	PET	3-modality	3-modality
Sensitivity (%)	56.7	50.0	30.0	60.0	36.7
Specificity (%)	76.0	81.3	97.3	92.0	100
Accuracy (%)	70.5	72.4	78.1	82.9	81.9

*nCECT* non-contrast-enhanced computed tomography, *CECT* contrast-enhanced computed tomography, *PET* positron emission tomography

## Discussion

In this study, the utility of a DL model in the diagnosis of lymph node metastasis was investigated. Consequently, a multimodal DL model was developed that integrates images from three different modalities: nCECT, CECT, and PET. This multimodal approach yielded a high AUC, demonstrating diagnostic performance comparable to that of experienced clinical experts.

This study utilized a multimodal learning approach incorporating two imaging modalities, CT and PET. Multimodal learning is considered to have enabled the DL model to effectively integrate information by capturing the complex biological characteristics of metastatic lymph nodes and the interrelationships among different imaging modalities, thereby potentially enhancing diagnostic performance. The utility of DL models incorporating multimodal imaging has been increasingly demonstrated in the recent literature. For example, contrast-enhanced multiphase CT images have been employed to differentiate malignant liver tumors [34], whereas multiple magnetic resonance imaging sequences, such as T1-weighted and T2-weighted images, along with clinical data, have been used to predict the therapeutic response to preoperative chemotherapy in patients with breast cancer [21]. By integrating the three modalities, the DL models demonstrated comparable performance to experienced experts. Furthermore, the multimodal DL model showed 60% sensitivity, which is substantially higher than the sensitivity of the expert assessments (~ 37%). This difference may be attributed to the inherent challenges in diagnosing lymph node metastasis in the absence of the uptake of ^18^F-FDG on PET as well as the limited diagnostic information provided by nCECT relative to CECT, which likely contributed to the lower sensitivity observed in expert-led evaluations. These findings suggest that the multimodal DL model may be capable of detecting lymph node metastases even in cases lacking the significant uptake of ^18^F-FDG on PET and/or contrast enhancement on CT.

This study was based on CT and PET images, both of which are routinely acquired during the preoperative evaluation of esophageal cancer. The developed DL model can be implemented in other clinical facilities by applying the same image-size standardization procedures utilized in this study. However, the quality and quantitative characteristics of CT and PET images may vary depending on the imaging equipment, acquisition protocols, and postprocessing conditions. Although standardization efforts are currently being promoted by relevant academic societies [35, 36], inter-institutional and inter-device variability may still influence the model performance. However, even if the DL model does not achieve optimal diagnostic performance at the initial stage of equipment installation in other institutions, its accuracy can be enhanced using a transfer-tuning approach. This method involves fine-tuning the pre-trained DL model developed in the present study using institution-specific imaging data, thereby enabling adaptation and performance improvement, even with relatively small datasets [37]. Moreover, unlike real-time DL applications, such as AI-based endoscopic diagnosis [38], the model presented in this study does not require an immediate image analysis. As a result, diagnostic evaluations may be conducted without the direct involvement of a radiologist, potentially facilitating a rapid and accurate pretreatment diagnosis. Given that the diagnostic performance of the system is comparable to that of experienced esophageal experts, it may facilitate an accurate diagnosis and appropriate treatment decisions, even in facilities with a limited number of esophageal cancer cases and restricted access to expert clinicians. Although the sensitivity of the three-modality DL model exceeded that of expert evaluations, it remained lower than the specificity demonstrated by the multimodal DL model. This discrepancy may be attributed to the smaller number of lymph nodes with metastasis relative to those without metastasis. In future studies, the sensitivity may be further improved by increasing the number of metastatic cases included in the training dataset.

Ongoing research on AI-based lymph node segmentation suggests that the integration of segmentation techniques with the DL model may enable the future development of a fully automated system capable of detecting lymph node metastasis directly from whole imaging datasets [39]. Furthermore, incorporating clinical parameters such as the depth of tumor invasion and tumor location, which are commonly used as decision-making criteria in clinical practice, may further contribute to improving the diagnostic accuracy of clinical staging.

The findings of this study are expected to provide a foundation for future research on multimodal DL models. Clinical staging based on DL models holds promise for enabling consistent and standardized diagnostic practices across all medical institutions. Such standardization is essential not only for determining appropriate treatment strategies at individual facilities, but also for ensuring objectivity, reliability, and reproducibility in multicenter collaborative studies. Accordingly, clinical staging driven by multimodal DL models may serve as a significant breakthrough to promote future advancements in the field of medicine. The present study represents a pivotal step toward the implementation of multimodal DL-assisted clinical staging.

This study had some limitations. First, lymph node metastasis was evaluated based on the pathological findings following surgical resection. As a result, discrepancies may have occurred in cases where metastases had been completely eradicated by preoperative chemotherapy, specifically, instances in which metastasis was present prior to treatment but undetectable in the resected specimens. Second, the lymph node diagnosis was conducted by manually extracting images centered on the lymph nodes, which may have introduced a selection bias. Third, CT and PET images were acquired using two different devices for each modality, potentially leading to variability in the image quality and quantitative characteristics. However, appropriate standardization procedures were applied; therefore, the impact on the results of this study was minimal.

This study is the first to demonstrate the utility of a DL model integrating three imaging modalities (nCECT, CECT, and PET) for the diagnosis of lymph node metastasis. The diagnostic performance of the multimodal DL model was comparable to that of experienced specialists in esophageal cancer. Multimodal DL model-based clinical staging offers the potential to provide consistent and standardized diagnoses across all medical institutions.

## Supplementary Information

Below is the link to the electronic supplementary material.

Supplementary file1 (XLSX 11 KB)
